# Integrated Physiotherapeutic Intervention for Rehabilitation of a Patient With Intellectual Disabilities: A Case Report

**DOI:** 10.7759/cureus.56476

**Published:** 2024-03-19

**Authors:** Mansi N Deshmukh, Pallavi Harjpal

**Affiliations:** 1 Department of Neuro Physiotherapy, Ravi Nair Physiotherapy College, Datta Meghe Institute of Higher Education and Research, Wardha, IND

**Keywords:** cognition, physical therapy, rehabilitation, physiotherapy, intellectual disability

## Abstract

Intellectual disabilities (ID) encompass a broad spectrum of neurodevelopmental disorders marked by impairments in cognitive functioning and adaptive behavior. Accessing and benefiting from rehabilitation services pose significant challenges for individuals within this population. In this case study, the rehabilitation journey of a 44-year-old man with ID, emphasizes the tailored approach to his rehabilitation program. The primary objectives of the program were to augment the patient's functional capabilities, foster independence, and enhance his overall quality of life. The case highlights the significance of personalized, comprehensive rehabilitation strategies intricately tailored to address the distinct requirements of individuals with ID. The case study delineates a comprehensive rehabilitation regimen integrating physical therapy to address the multifaceted needs of individuals with varying degrees of disability. This inclusive approach represents a paradigm shift toward a multidisciplinary (physiotherapy along with general medical care, special education, vocational training, and community-based interventions) person-centered model of care. Through addressing the varied needs of individuals with ID, the rehabilitation plan endeavors to empower them to lead enriching, self-directed lives within their communities, thereby unlocking their complete potential. This case study stands as evidence of the profound impact of customized rehabilitation interventions in cultivating inclusivity and optimizing the well-being of individuals with ID.

## Introduction

Intellectual disability (ID) is a multifaceted condition marked by cognitive limitations and deficiencies in adaptive functioning, emerging during the early developmental stages [1]. In adults, the manifestation of aggressive and confrontational behaviors is commonplace among those with intellectual disabilities, management through the administration of antipsychotic medications becomes one of the strategies along with various others [2]. Management strategies for individuals with intellectual impairments encompass a multidisciplinary approach, including behavioral symptom management, treatment of comorbid conditions, general medical care, special education, vocational training, and community-based interventions. Physical activity holds particular importance for the physical and psychological well-being of youth with intellectual impairments. While weight training demonstrates significant physical benefits, engagement in sports activities also enhances both mental and physical health outcomes [3]. The etiology of intellectual disabilities spans from genetic mutations to environmental factors, with hereditary components implicated in up to 40% of cases [4]. As per the American Association on Intellectual and Developmental Disabilities, individuals diagnosed with ID display notable restrictions in intellectual capacities (encompassing reasoning, learning, and problem-solving) as well as adaptive behavior (spanning conceptual, social, and practical skills), often emerging prior to reaching adulthood, typically before the age of 18 [5].

In England, the prevalence rates of ID are reported at 2.7% among school-aged children and 2.17% among adults [6]. Legislative measures, such as Rosa's Law (Public Law 111-256), have replaced the term "mental retardation" with "intellectual disability" [7]. ID can be classified as either genetic or non-genetic, with genetic factors contributing to 30% to 50% of cases. Genetic causes include single gene disorders (e.g., Prader-Willi syndrome), inherited genetic traits (e.g., fragile X syndrome), and chromosomal abnormalities (e.g., trisomy 21 syndrome) [8]. Current evidence suggests that antipsychotic medications may alleviate problematic behaviors in children with intellectual impairments in the short term [9].

## Case presentation

Patient information

A 44-year-old male patient presented to our hospital with prominent symptoms of cough, cold, and fever, concomitant with behavioral aberrations. The patient demonstrates profound social withdrawal, exhibiting monosyllabic speech and occasional wandering tendencies. He exhibits an inability to perform basic activities of daily living and lacks control over bowel and bladder functions. Additionally, he has ceased eating independently for the past seven days. Despite comprehending commands, the patient does not engage in verbal communication. He carries a diagnosis of ID, with no reported familial history of the condition. While conscious, the patient displays disorientation concerning time, place, and personal identity. Admission to the hospital occurred on September 21, 2023, with a subsequent referral for physiotherapy on September 23, 2023.

Clinical findings

Upon obtaining consent, a comprehensive examination of the patient was conducted. Observations indicate persistent disorientation regarding time, place, and personal identity. The examination was performed with the patient in a supine position. Vital signs including blood pressure (110/70 mmHg) and pulse rate (72 bpm) were within normal limits. Manual muscle testing was conducted and findings are summarized in Table 1. Assessment of muscle tone utilized a tone grading scale, with results detailed in Table 2. Reflexes were evaluated and are presented in Table 3.

**Table 1:** Manual muscle testing on assessment 2/5: Full range of motion gravity eliminated; 3/5: Full range of motion against gravity

Manual muscle testing	Movement	Right	Left
Shoulder	Flexors	3/5	3/5
	Extensors	3/5	3/5
Elbow	Flexors	3/5	3/5
	Extensors	3/5	3/5
Wrist	Flexors	3/5	3/5
	Extensors	3/5	3/5
Hip	Flexors	2/5	3/5
Knee	Flexors	3/5	3/5
	Extensors	3/5	3/5
Ankle	Plantar flexors	3/5	3/5
	Dorsiflexors	3/5	3/5

**Table 2:** Muscle tone on assessment 2+: Normal response

Muscle tone	Right	Left
Shoulder	2+	2+
Elbow	2+	2+
Wrist	2+	2+
Hip	2+	2+
Knee	2+	2+
Ankle	2+	2+

**Table 3:** Reflexes ++: Normal

Reflexes	Biceps	Triceps	Supinator	Knee	Ankle	Plantar response
Right	++	++	++	++	++	Flexors
Left	++	++	++	++	++	Flexors

Therapeutic interventions

Table 4 shows the therapeutic interventions administered over a four-week period, accompanied by the recording of pre- and post-treatment outcomes.

**Table 4:** Physiotherapy protocol from week one to week four PNF: Proprioceptive neuromuscular facilitation; ROM: Range of motion; AROM: Active range of motion; reps: Repetitions; mins: Minutes

Goal	Intervention	Duration
Week 1		
To improve ROM	AROM for shoulder joint, elbow joint, hip joints in all planes	10 reps
To improve strength	Isometrics strengthening exercise for hip and knee joint, upper limb strengthening exercise	10 reps
To improve joint stability	PNF pattern (hold relax)	10 reps
Week 2		
To improve ROM	AROM for shoulder joint, elbow joint, hip joint in all planes	10 repetitions, 3 times per day
To improve strength	Isometrics strengthening exercises for hip and knee joints, upper limb strengthening exercise	10 reps
To improve joint stability	PNF pattern (hold relax)	10 reps
To improve gait pattern	Parallel bar walking	2 rounds
To improve task performance	Task-specific practice	10 mins
Week 3		
To improve ROM	AROM for shoulder joint, elbow joint, hip joints in all planes	10 reps
To improve strength	Isometrics strengthening exercise for hip and knee joint, upper limb strengthening exercise	10 reps
To improve joint stability	PNF pattern (hold relax)	10 reps
To improve gait pattern	Parallel bar walking	3 rounds
To improve task performance	Task-specific practice [10]	10 mins
To increase strength and physical activity level	Progressive resistance training	10 reps with 1 kg weight cuff
To improve balance, gait pattern, and improve physical activity	Treadmill walking [11]	5 mins
Week 4		
To improve ROM	AROM for shoulder joint, elbow joint, hip joints in all planes	10 reps
To improve strength	Isometrics strengthening exercise for hip and knee joint, upper limb strengthening exercise	10 reps
To improve joint stability	PNF pattern (hold relax)	10 reps
To increase strength and physical activity level	Progressive resistance training	10 reps with 2 kg weight cuff
To improve gait pattern	Parallel bar walking	4 rounds
To improve gait training	Obstacle walking	2 rounds
To improve task performance	Task-specific practice	10 mins
To improve balance, gait pattern, and improve physical activity	Treadmill walking [12]	5 mins
Increase proprioception, sensory awareness	Use of trampoline [13]	5 mins

**Figure 1:**
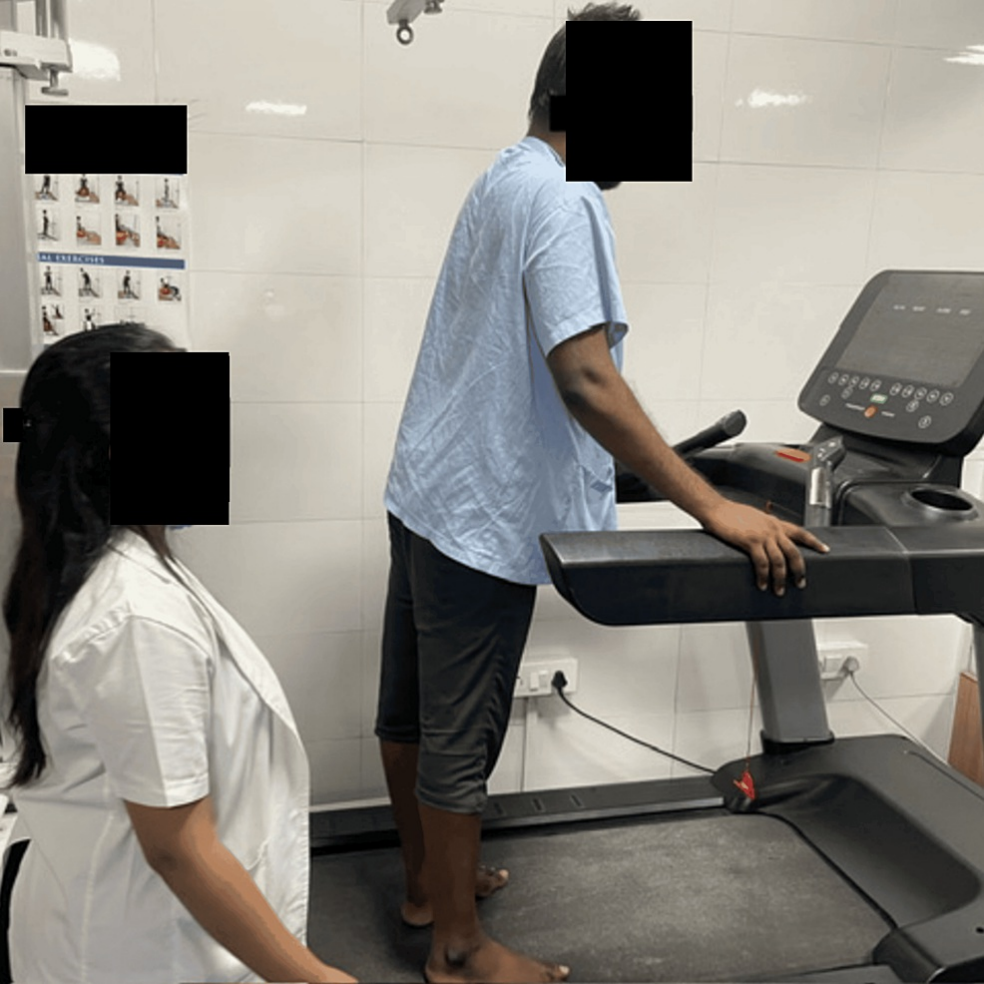
Treadmill walking for the patient with intellectual disability

**Figure 2:**
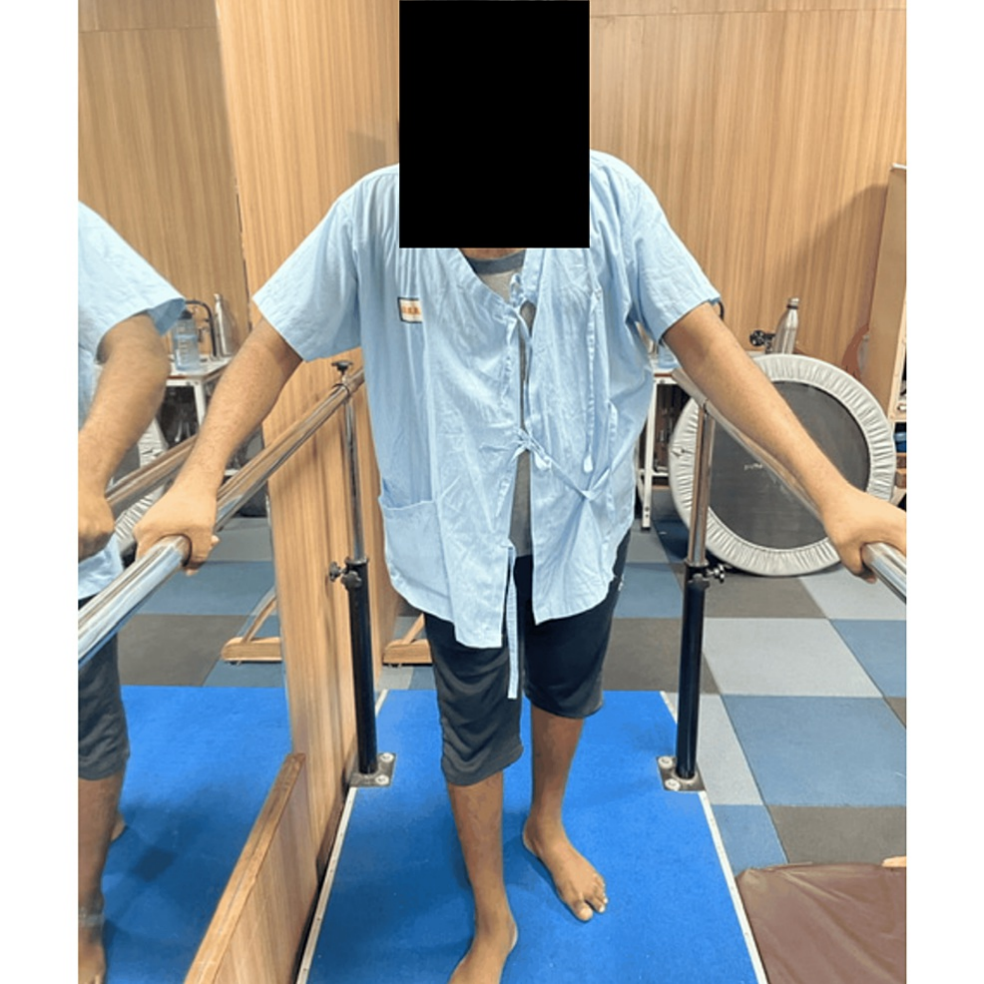
Parallel bar walking for the patient with intellectual disability

Figures 1, 2 visually depict the patients undergoing the specified treatment procedures.

Outcome measure

Table 5 shows outcome measures both before and after a four-week rehabilitation protocol. Subsequently, assessments were conducted using the Berg Balance Scale, Functional Reach Test, and Barthel Index. The ensuing results are presented below.

**Table 5:** Outcome measures before and after the rehabilitation BBS: Berg balance scale

Outcome measure	Pre-rehabilitation	Post-rehabilitation
BBS	16/56	44/56
Functional reach test	2 inches	7 inches
Barthel index	18/100	75/100

## Discussion

In this case report, physiotherapy emerges as a pivotal element in expediting the patient's recovery process. The intervention proved instrumental in enhancing mobility, augmenting strength, refining gait patterns, and facilitating the patient's performance of task-specific activities and ultimately enhanced the quality of life by improving activities of daily living. ID encompasses deficits in both cognitive functioning and adaptive behavior, typically manifesting from infancy through adulthood. Crafting a physical rehabilitation program tailored to individuals with intellectual disabilities necessitates a comprehensive, patient-centric approach. Recognizing the unique needs, abilities, and constraints of each individual is paramount.

Physical therapists employ diverse strategies to address intellectual disabilities. For instance, treadmill exercises among adolescents with intellectual disabilities have demonstrated enhancements in walking ability, alongside reductions in blood pressure and oxidative stress, while concurrently improving gait patterns [14]. Progressive resistance training has shown promise in augmenting strength and potentially elevating levels of physical activity among children and adolescents with intellectual disabilities [15]. Task-specific practice interventions further facilitate the generalization of skill performance through extended, repetitive practice sessions. Exercise therapy interventions yield positive physical outcomes for individuals with intellectual disabilities, including improvements in muscular strength [16].

## Conclusions

A comprehensive and individualized physiotherapy rehabilitation approach is imperative for patients with intellectual disabilities. The primary objectives encompass enhancing overall well-being, fostering independence, and optimizing functional capacities. This multifaceted strategy may entail rigorous assessment and diagnosis, targeted social skills development, therapeutic interventions, and the active participation of both family members and caregivers in therapeutic exercises and gait training. It is crucial to underscore that the success of rehabilitation in individuals with intellectual impairments hinges significantly upon the practitioner's approach, patience, and consistent application of therapeutic modalities. These essential components collectively contribute to the efficacy of rehabilitation efforts, ensuring a holistic and tailored intervention for patients with intellectual disabilities and greater outcomes.
